# Pulmonary Vein Isolation with a Novel Size-Adjustable Cryo-Balloon Catheter: A Tailored Ablation Protocol

**DOI:** 10.3390/jcm13082262

**Published:** 2024-04-13

**Authors:** Yannick Teumer, Franziska Hilgarth, Lyuboslav Katov, Rima Melnic, Wolfgang Rottbauer, Carlo Bothner, Karolina Weinmann

**Affiliations:** Department of Cardiology, Ulm University Heart Center, Albert-Einstein-Allee 23, 89081 Ulm, Germany; yannick.teumer@uniklinik-ulm.de (Y.T.);

**Keywords:** atrial fibrillation, pulmonary vein isolation, cryo-balloon, size-adjustable cryo-balloon, PolarX Fit

## Abstract

**Background:** Pulmonary vein isolation (PVI) is a common therapeutic approach for symptomatic atrial fibrillation (AF). Among various techniques, cryo-balloon (CB) PVI is widely adopted, but, to date, established CB systems have had fixed balloon sizes. A novel size-adjustable CB, allowing balloon size adjustments during ablation, lacks sufficient data on optimal utilization in patient care. This study aims to systematically investigate this feature with a tailored ablation protocol. **Methods:** Our single-center prospective study included patients with paroxysmal or persistent atrial fibrillation undergoing first-time PVI with the size-adjustable CB from July 2023 to February 2024. Ablation was performed using the balloon size that provided better occlusion. The ablation protocol involved an initial occlusion test with the small balloon size (28 mm). If optimal occlusion (occlusion level 4) could not be achieved, an attempt with the larger balloon (31 mm) was initiated. Ablation was conducted using the balloon configuration that provided better occlusion of the pulmonary vein ostium. **Results:** Our prospective study includes 50 patients (median age [interquartile range, IQR]: 72 [65; 79] years, 24 [48.0%] females, and 35 [70.0%] patients with paroxysmal AF). The median procedure duration (IQR) was 77 (65; 96) minutes, and the median fluoroscopy time (IQR) was 17.7 (12.5; 22.0) min. PVI was successfully accomplished in each treated pulmonary vein (PV), with 87.4% of PVs isolated during the first freeze. The large balloon configuration was used to isolate 16.8% of PVs. **Conclusions:** The utilization of the size-adjustable CB, combined with the presented tailored ablation workflow, appears to facilitate effective and efficient pulmonary vein isolation. The use of a larger balloon configuration appears beneficial in isolating a significant proportion of the PVs.

## 1. Introduction

Atrial fibrillation (AF) stands as the most prevalent sustained cardiac arrhythmia [1,2], and its incidence is anticipated to rise in the years ahead [1,2,3]. In addition to addressing thromboembolic events and managing AF risk factors, effective symptom control plays a pivotal role in its treatment [1,2]. Symptom control can be attained through either frequency or rhythm control methods [1,2]. While electrical cardioversion and antiarrhythmic drug therapy are options for rhythm control, atrial fibrillation ablation is an increasingly utilized method.

The primary trigger for atrial fibrillation is widely recognized to be ectopic beats originating from the pulmonary veins [4]. Consequently, the cornerstone of an AF ablation procedure is pulmonary vein isolation (PVI) [1,2]. According to the currently valid AF guidelines of the American Heart Association and the European Society of Cardiology, PVI can be a first-line therapy for patients with symptomatic atrial fibrillation [1,2]. This approach aims to diminish recurrence and enhance the quality of life by reducing the burden of atrial fibrillation [1,2], especially in symptomatic patients with paroxysmal AF, where PVI outperforms drug therapy in preventing recurrence [5].

PVI can be achieved through various ablation systems utilizing different types of ablation energy. The most used PVI modalities are point-by-point radiofrequency and cryo-balloon (CB) PVI. In comparison to point-by-point radiofrequency PVI, CB-PVI is deemed non-inferior in terms of efficiency and safety [6]. However, all currently available balloon catheters have a fixed balloon size that cannot be adjusted during the procedure, except by changing the catheter. 

Recently, a novel size-adjustable CB system was introduced, allowing adjustment of the balloon size during the procedure. With a simple touch of a button, the novel size-adjustable CB can transition from a small balloon configuration (28 mm diameter) to a large balloon configuration (31 mm diameter). Currently, there is a lack of data on how this novel size adjustment feature should be utilized in patient care. Furthermore, evidence regarding procedural data and the safety profile of the novel size-adjustable CB system is limited. This prospective monocentric study aims to systematically investigate the novel size adjustment feature in conjunction with a tailored ablation protocol.

## 2. Materials and Methods

### 2.1. Study Population

In this prospective registry study, patients with symptomatic atrial fibrillation were enrolled between July 2023 and February 2024. Patients with paroxysmal or persistent AF and a scheduled first-time PVI-only procedure at Ulm University Medical Center, Ulm, Germany were included in our study. Exclusion criteria were long-standing persistent AF, a left atrial (LA) diameter larger than 55 mm, and patients younger than 18 years. All patients underwent a first-time PVI-only procedure with the novel size-adjustable cryo-balloon catheter. The data were collected prospectively as part of the ATRIUM registry (German Clinical Trials Register-ID: DRKS00013013). Each patient gave written informed consent. The study received approval from the local Ethics Committee at Ulm University and adhered to the principles of the Declaration of Helsinki.

### 2.2. Components and Characteristics of the Ablation System

**Figure 1:**
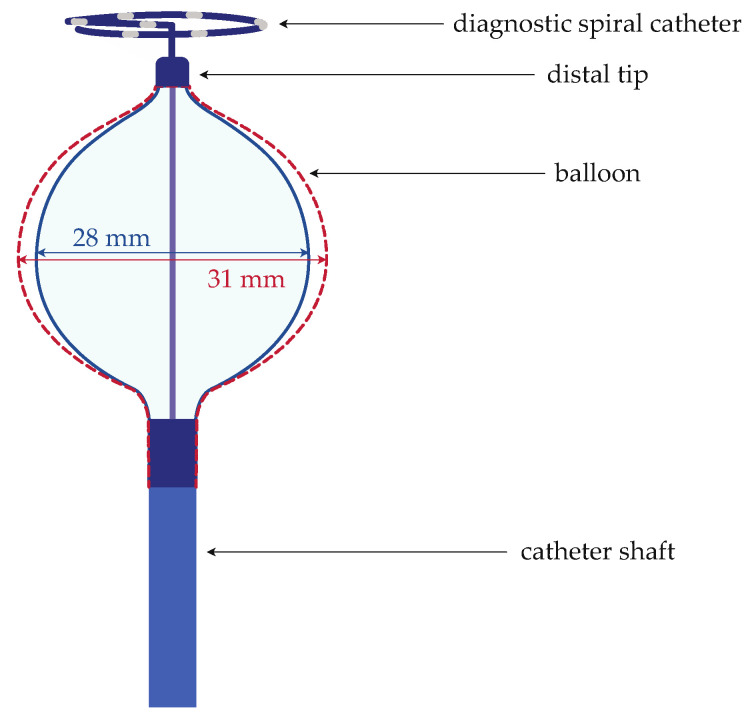
Schematic depiction of the novel size-adjustable cryo-balloon catheter. The figure illustrates the novel size-adjustable cryo-balloon catheter in both the small configuration (28 mm diameter, depicted with a blue continuous line for the balloon shape) and the large configuration (31 mm diameter, depicted with a red dashed line for the balloon shape).

In this study, a novel, compliant and size-adjustable CB catheter (PolarX Fit, Boston Scientific, Marlborough, MA, USA, Figure 1) was used. The size of this CB can be adjusted during the procedure and inside the patient by pressing a button. The different balloon sizes of 28 mm and 31 mm originate in a different pressure inside the balloon. Similar to other standard CB systems, the balloon is positioned at the vein via a diagnostic spiral catheter (Polarmap, Boston Scientific, Marlborough, MA, USA, Figure 1), the shaft of which passes through the inner lumen of the balloon catheter. This spiral catheter contains eight electrodes to detect pulmonary vein (PV) signals during the freezes and is the same catheter as in the standard, non-size-adjustable PolarX system.

### 2.3. Periprocedural Management, Ablation Procedure, and Postprocedural Management

All procedures were performed under uninterrupted oral anticoagulation. Antiarrhythmic drugs were discontinued before the procedure. Deep sedation was uniformly attained in all patients throughout the procedure, adhering to the standardized local sedation protocol [7], which entails the administration of midazolam, propofol, and fentanyl. To monitor esophageal temperature, a multi-electrode temperature probe (S-Cath, Circa Scientific LLC, Englewood, CO, USA) was trans-nasally placed into the esophagus at the level of the left atrium in each patient according to the local standards.

Two femoral vein punctures were performed under ultrasound guidance. A steerable diagnostic decapolar catheter (Inquiry, 6F, Abbott, Chicago, IL, USA) was placed in the coronary sinus for electrogram recording. Temporarily, the decapolar catheter was placed into the superior vena cava for phrenic nerve pacing during the freeze in the right sided PVs. A transesophageal echocardiography (TEE) was performed to rule out left atrial thrombus. After transseptal puncture under fluoroscopic and transesophageal echo guidance (HeartSpan Transseptal Needle, Biosense Webster, Irvine, CA, USA; Cardia Guide Fixed Sheath, Biosense Webster, Irvine, CA, USA), an unfractionated heparin bolus was administrated, targeting an activated clotting time between 300 and 350 s during the procedure. 

After the transseptal puncture, the steerable sheath (PolarSheath, 15.9 Fr, Boston Scientific, Marlborough, MA, USA) of the cryoablation system was placed in the LA. Selective PV angiography was accomplished to identify all PV ostia via the steerable sheath. Then, the CB (PolarX Fit, Boston Scientific) with the intraluminal diagnostic spiral catheter was placed in the LA. PV occlusion was guided by contrast injection.

During the freeze, the PV electrograms were monitored on the diagnostic spiral catheter for time-to-isolation (TTI) observation. A PVI after a single freeze was defined as single-shot isolation. During the ablation of the right PV, the phrenic nerve was stimulated, and the diaphragm contraction was monitored to observe the phrenic nerve function. After catheter removal, a Z-suture was applied at the puncture site, and additional manual compression was performed for 5–10 min. A groin pressure bandage was applied at the puncture site for 6 h, and after 6 h, the Z-suture was removed, followed by the application of a groin pressure bandage for an additional 6 h. Echocardiography ruled out pericardial effusion immediately after the procedure. Patient vital parameters were monitored for 24 h, including clinical examination, transthoracic echocardiography, and a 12-lead resting electrocardiogram (ECG). 

### 2.4. Tailored Ablation Protocol

The tailored ablation protocol is based on our TTI-based ablation protocol, which has been described in detail before [8]. In summary, the duration of the freeze and the application of protentional bonus freezes depended on the TTI observation in each PV. If the TTI ranged between 30 and 60 s, a single 180-s freeze was administered. If the TTI was less than 30 s, the freeze duration was reduced to 120 s. If the TTI exceeded 60 s, or the TTI could not be observed but pulmonary vein (PV) isolation was successful, after a 180-s freeze, an additional 180-s bonus freeze was applied.

In addition to the original TTI-based ablation protocol, the balloon size used for ablation depended on the individual occlusion of the pulmonary vein. The first step was an occlusion attempt with the small balloon configuration (28 mm). If optimal occlusion (occlusion level 4 = complete occlusion) was reached, the ablation was performed with the small balloon. If optimal occlusion (occlusion level < 4) was not achieved with the small balloon configuration, an occlusion attempt with the large balloon configuration (31 mm) was performed. If optimal occlusion was reached (occlusion level 4), the ablation was therefore performed with the large balloon configuration. If neither with the small nor the large balloon optimal occlusion was possible, the ablation was performed with the balloon configuration with the better occlusion level. A switch of the balloon size was possible if no isolation was achieved with the chosen balloon configuration. 

**Figure 2:**
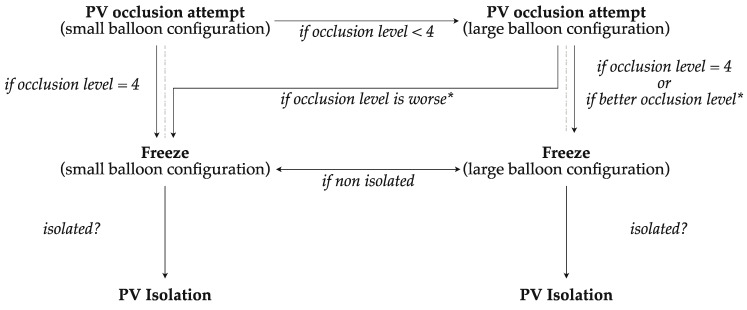
Flowchart for the selection of balloon sizes for pulmonary vein isolation with the novel size-adjustable cryo-balloon. PV, pulmonary vein; * in comparison to the occlusion level with the small balloon configuration.

Furthermore, the ablation protocol stipulates that, in the case of a significant esophageal temperature drop during the freeze and premature termination of the ablation, the next freeze should be performed with the large balloon configuration (Figure 2).

### 2.5. Statistical Analysis

Statistical analysis was performed using SPSS Statistics (version 29.0.1.0, IBM, Armonk, NY, USA). Categorical variables were presented as absolute and relative proportions. Continuous variables were tested for normal distribution. Normally distributed variables were expressed as mean ± standard deviation. Non-normally distributed variables were described as median with interquartile range (IQR).

## 3. Results

### 3.1. Patient Characteristics

In this study, 50 patients (48% female) were included. The median (IQR) age of the patients was 72 (65; 79) years, and they had a median CHA2DS2-VASc Score of four (three; five). Of the participants, 35 patients (70.0%) were ablated cause of paroxysmal AF. More detailed patient characteristics are summarized in Table 1. 

Before the indication for catheter ablation was given, 41 patients (82.0%) received β-blockers, and 7 patients (14.0%) received antiarrhythmic drugs (class Ic: *n* = 2 (4.0%); class III: 5 (10.0%)).

### 3.2. Procedural Characteristics and Ablation Data

The median procedure duration (IQR) was 77 (65; 96) minutes, the median dwell time (IQR) was 41 (32; 55) minutes, and the median fluoroscopy time was 17.7 (12.5; 22.0) minutes. Deviating from the typical anatomy with two left and two right PVs, a total of six patients showed an anomalous anatomy. Five patients (10.0%) had a common ostium in the left sided pulmonary veins, and one patient (2.0%) had an additional right vein (RMPV, right middle pulmonary vein, Table 2). PVI was achieved in every treated PV (*n* = 196, 100%). Single-shot isolation was possible in 171 of 196 (87.2%) PVs. The TTI was observed in 72.4% of all treated PVs.

In 27 PVs (13.8%), a better occlusion could be achieved with the larger balloon configuration. It should be emphasized that all five LCPVs were isolated in the PV ostium with the larger balloon configuration. The initially selected balloon configuration was switched twelve times (6.1%) before achieving PVI (Table 3). 

The reasons for the mixed balloon size in the same PV varied. Five times, a switch from the small to the large balloon configuration was performed due to unsuccessful isolation with the small balloon configuration, despite initial good PV occlusion with the small balloon configuration. Conversely, the balloon configuration was changed six times from large to small due to unsuccessful isolation, despite initially better occlusion of the PV with the larger balloon configuration. A switch in the balloon size from small to large due to a freeze abortion because of a too low esophageal temperature happened once in the LIPV. Table 4 provides the reasons for balloon size switch per vein.

### 3.3. Safety

There were no periprocedural fatalities, vascular access complications, pericardial effusion, tamponade, persistent phrenic nerve palsy, transient ischemic attacks, or strokes observed. In two cases, a transient phrenic nerve palsy (PNP) occurred. One transient PNP occurred during the ablation on the RSPV, another one during the ablation in the RIPV. The freezes were stopped immediately, and in both cases, phrenic nerve palsy resolved spontaneously by the end of the procedure.

## 4. Discussion

To the best of our knowledge, this study is the first to describe an ablation protocol that systematically uses the possibility of resizing the novel CB according to the individual PV anatomy. Furthermore, evidence concerning the procedural data and the safety of the novel size-adjustable CB system is sparse. 

### 4.1. Efficacy

Despite a potential additional step in the procedure, in terms of adjusting the balloon size, the total procedure time of the novel size-adjustable CB system is similar compared to other non-size-adjustable CB systems [9,10,11,12] for PVI. In our view, this suggests that the size-adjustable CB system can be time-efficient, despite an additional procedure step.

The novel size-adjustable CB system appeared to be particularly helpful in unexpected special anatomical and procedural conditions, for example, in patients with common ostia or significant esophageal temperature drop during the freeze. All common ostia in this study were better occluded by the larger balloon configuration. Interestingly, single shot isolation was possible with the larger balloon configuration in three of five (60.0%) treated LCPVs. In one patient, a significant esophageal temperature drop was recognized during the freeze in the LIPV. After changing the balloon size from small to large, a 24 s longer freeze, and thus the isolation of the PV, was possible. In the event of an esophageal temperature drop, the larger balloon configuration appeared to facilitate prolonged freezing. During ablation with the small balloon configuration, the ablation is typically performed deeper in the vein compared to the larger balloon configuration [13]. In theory, ablation with the larger balloon configuration shifts the ablation site more into the atrium, creating a greater distance between the ablation site and the individual position of the esophagus, thus allowing for a longer freeze. The potential benefit of size-adjustability in CB-PVI in combination with the presented tailored ablation protocol is further emphasized by this. 

The size-adjustable CB may offer additional advantages in patients with reduced ejection fraction and increased left atrial volumes. Since the PV ostia expand with the diameter of the left atrium, the innovative CB could broaden the range of patients suitable for treatment with the single-shot device, as the balloon diameter remains compatible with the PV diameter. However, due to the limited number of patients with reduced ejection fraction, one can only speculate about potential benefits.

The fluoroscopy time was slightly longer than in other studies performed with standard non-size-adjustable CB systems [9,10,12,14,15]. An explanation for the difference could be that, in the case of non-optimal PV occlusion with the small balloon configuration, it was possible to switch to the larger balloon configuration. The adjustment of the balloon size and the additional needed occlusion attempts with the enlarged balloon were performed under fluoroscopic guidance. This may lead to a slightly longer fluoroscopy time with the size-adjustable CB system. Further investigations are necessary in this regard.

The total left-atrial dwell time with the novel size-adjustable CB system was numerically shorter than in other PVI studies with a non-size-adjustable CB system [9,14]. Furthermore, in our study, the total PV isolation rate was relatively high, compared to the non-size-adjustable CB systems [9,10,12,14,15]. A numerically shorter left atrial dwell time and a relatively high overall PVI rate may suggest that the size-adjustability, in conjunction with our tailored ablation protocol, could enhance the efficiency of CB-PVI. 

Furthermore, CB-PVI is now in competition with pulsed field ablation, a single-shot device utilizing non-thermal energy and devoid of esophageal or phrenic nerve side effects [16,17]. Whether the size-adjustable CB confers an advantage over pulsed field ablation necessitates evaluation through scientific assessment.

According to our protocol, the large balloon configuration was used in every fifth PV in our study. Thereby, 16.8% of all treated PV were isolated using the large balloon configuration. A potential benefit of size-adjustability in CB-PVI is further emphasized by this. 

In the FIT extension arm of the FROZEN-AF trial, in 64% of the PV, the larger balloon configuration was utilized at the discretion of the interventionalist [13]. In numerical terms, the large balloon configuration was therefore used around 3.8 times more frequently in this study than with our protocol. However, it must be mentioned at this point that the larger balloon configuration caused the ablation line to move closer together, especially at the posterior wall of the left atrium between the right and left PVs. In our view, unsystematic use of the large balloon configuration can therefore lead to the creation of a critical isthmus, especially in small left atria. The induction of secondary atrial tachycardia is therefore conceivable, so that in our view the large balloon configuration should not be used uncritically and unsystematically. 

### 4.2. Safety

No major complications occurred in this study using the novel size-adjustable CB system and our ablation protocol. In particular, with regard to the PNP rate, the novel size-adjustable CB system is comparable with the data available in the literature on the established non-size-adjustable CB systems [18,19,20]. Unexpectedly, there were no complications related to vascular access. This may be attributed to the use of sonographic guidance for femoral venous punctures and is not correlated to the size-adjustable CB or our ablation protocol, in our opinion. 

However, to verify the benefit of the size adjustability, a direct comparison must be made between the size-adjustable and non-size-adjustable CB systems. 

### 4.3. Limitations

The availability of a size-adjustable CB provides interventionalists with an additional tool to address unforeseen anatomical variations. While the findings remain promising, it is imperative to acknowledge the limitations inherent in the study, namely the restricted sample size and the single-center design. To mitigate these limitations, future research endeavors should prioritize multi-center, randomized trials to conclusively validate the hypotheses generated.

## 5. Conclusions

An effective isolation of pulmonary veins is achievable through the utilization of the size-adjustable CB in conjunction with the tailored ablation protocol presented. Overall, the procedural data using this size-adjustable CB seem to be comparable to other non-size-adjustable CB systems. In combination with a tailored ablation protocol, this potentially additional procedure step does not seem to have a relevant negative impact on the overall procedure duration. The novel size-adjustable CB system enhances the examiner’s flexibility in addressing non-standard anatomy and potential procedural issues, such as a significant esophageal temperature drop during ablation, by incorporating a bail-out strategy through the size adjustment feature.

## Figures and Tables

**Table 1:** Baseline characteristics of the patients treated with the novel size-adjustable cryo-balloon.

Baseline Characteristics	Total (*n* = 50)
Age [years], median (IQR)	72 (65; 79)
Female, *n* (%)	24 (48.0%)
BMI [kg/m^2^], median (IQR)	26.7 (23.9; 30.8)
Paroxysmal AF, *n* (%)	35 (70.0%)
CHA_2_DS_2_-VASc score, median (IQR)	4 (3; 5)
LA diameter [mm], median (IQR)	45 (42; 50)
LAVI [mL/m^2^], median (IQR)	42 (35; 58)
LVEF (%), median (IQR)	59 (46; 65)
Hypertension (*n*, %)	37 (74.0%)
Diabetes mellitus (*n*, %)	5 (10.0%)
Previous stroke/TIA (*n*, %)	6 (12.0%)
Coronary artery disease (*n*, %)	25 (50.0%)

AF, atrial fibrillation; BMI, body mass index; IQR, interquartile range; LA, left atrial; LAVI, left atrial volume index; LVEF, left ventricular ejection fracture; TIA, transient ischemic attack.

**Table 2:** Pulmonary vein ablation data.

Treated PVs (*n* = 196)	
Single-shot-isolation, *n* (%)	171 (87.2%)
Freezes per patient (including bonus freezes), median (IQR)	6 (6; 8)
Freezes per patient (without bonus freezes), median (IQR)	4 (4; 6)
LSPV (*n* = 45)	
Single-shot-isolation, *n* (%)	41 (91.1%)
TTI [s], median (IQR)	43 (34; 60)
Freezes per vein, median (IQR)	1 (1; 2)
LIPV (*n* = 45)	
Single-shot-isolation, *n* (%)	40 (88.9%)
TTI [s], median (IQR)	37 (27; 50)
Freezes per vein, median (IQR)	2 (1; 2)
RIPV (*n* = 50)	
Single-shot-isolation, *n* (%)	43 (86.0%)
TTI [s], median (IQR)	54 (33; 69)
Freezes per vein, median (IQR)	2 (1; 2)
RSPV (*n* = 50)	
Single-shot-isolation, *n* (%)	43 (86.0%)
TTI [s], median (IQR)	36 (30; 64)
Freezes per vein, median (IQR)	2 (1; 2)
LCPV (*n* = 5)	
Single-shot-isolation, *n* (%)	3 (60.0%)
TTI [s], median (IQR)	30.0 (- ^1^)
Freezes per vein, median (IQR)	3 (2; 4)
RMPV (*n* = 1)	
Single-shot-isolation, *n* (%)	1 (100.0%)
TTI [s]	20
Freezes per vein	1

IQR, interquartile range; LCPV, left common pulmonary vein; LIPV, left inferior pulmonary vein; LSPV, left superior pulmonary vein; PV, pulmonary vein; LCPV, left common pulmonary vein; RIPV, right inferior pulmonary vein; RMPV, right medial pulmonary vein; RSPV, right superior pulmonary vein; TTI, time to isolation. ^1^ Calculation of the IQR not reasonable due to the number of cases.

**Table 3:** Balloon size used for pulmonary vein isolation with the novel size-adjustable cryo-balloon.

	Small Balloon Only *n* (%)	Large Balloon Only *n* (%)	Mixed Balloon Size *n* (%)
treated PVs (*n* = 196)	157 (80.1%)	27 (13.8%)	12 (6.1%)
LSPV (*n* = 45)	37 (82.2%)	5 (11.1%)	3 (6.7%)
LIPV (*n* = 45)	38 (84.4%)	3 (6.7%)	4 (8.9%)
RIPV (*n* = 50)	39 (78.0%)	8 (16.0%)	3 (6.0%)
RSPV (*n* = 50)	42 (84.0%)	6 (12.0%)	2 (4.0%)
LCPV (*n* = 5)	0 (0%)	5 (100.0%)	0 (0%)
RMPV (*n* = 1)	1 (100.0%)	0 (0%)	0 (0%)

LCPV, left common pulmonary vein; LIPV, left inferior pulmonary vein; LSPV, left superior pulmonary vein; PV, pulmonary vein; LCPV, left common pulmonary vein; RIPV, right inferior pulmonary vein; RMPV, right medial pulmonary vein; RSPV, right superior pulmonary vein.

**Table 4:** Overview of the reasons for a mixed balloon size during the ablation in the same pulmonary vein.

	Small to Large Configuration		Large to Small Configuration
	Not Isolated PV	Esophagus Temperature Drop	Not Isolated PV
PVs overall, *n* = 12 (6.1%)	5	1	6
LSPV, *n* = 3 (1.5%)	1	0	2
LIPV, *n* = 4 (2.0%)	2	1	1
RIPV, *n* = 3 (1.5%)	2	0	1
RSPV, *n* = 2 (1.0%)	0	0	2

LIPV, left inferior pulmonary vein; LSPV, left superior pulmonary vein; PV, pulmonary vein; RIPV, right inferior pulmonary vein; RSPV, right superior pulmonary vein.

## Data Availability

The data presented in this study are available on request from the corresponding author. The data are not publicly available due to data privacy laws.
